# Monitoring drug promiscuity over time

**DOI:** 10.12688/f1000research.5250.2

**Published:** 2014-11-04

**Authors:** Ye Hu, Jürgen Bajorath

**Affiliations:** 1 Department of Life Science Informatics, B-IT, LIMES Program Unit Chemical Biology and Medicinal Chemistry, Rheinische Friedrich-Wilhelms-Universität, Bonn, D-53113, Germany

## Abstract

Drug promiscuity and polypharmacology are much discussed topics in pharmaceutical research. Experimentally, promiscuity can be studied by profiling of compounds on arrays of targets. Computationally, promiscuity rates can be estimated by mining of compound activity data. In this study, we have assessed drug promiscuity over time by systematically collecting activity records for approved drugs. For 518 diverse drugs, promiscuity rates were determined over different time intervals. Significant differences between the number of reported drug targets and the promiscuity rates derived from activity records were frequently observed. On the basis of high-confidence activity data, an increase in average promiscuity rates from 1.5 to 3.2 targets per drug was detected between 2000 and 2014. These promiscuity rates are lower than often assumed. When the stringency of data selection criteria was reduced in subsequent steps, non-realistic increases in promiscuity rates from ~6 targets per drug in 2000 to more than 28 targets were obtained. Hence, estimates of drug promiscuity significantly differ depending on the stringency with which target annotations and activity data are considered.

## Introduction

Promiscuous compounds specifically interact with multiple biological targets ^1^. As such, they are distinct from compounds that exhibit assay liabilities or engage in various non-specific interactions. Compound promiscuity is often functionally relevant and represents the molecular origin of polypharmacology ^2^, a concept that experiences increasing interest in drug discovery. Drugs are often, but not always, found to act on multiple targets and modulate multiple cellular pathways and/or signaling cascades. Such effects might often substantially contribute to therapeutic efficacy, for example, in cancer treatment ^3^. The potentially far reaching consequences of drug polypharmacology for therapy, the frequency of these effects, and likely pros and cons are just beginning to be understood.

Experimentally, promiscuity can be assessed by profiling of compounds or drugs on arrays of biological targets ^1, 2^, although such studies might often only provide an incomplete picture of *in vivo* effects. The same applies to computational estimates of promiscuity. Given the increasingly large amounts of compound activity data that are becoming available, the promiscuity of drugs and bioactive compounds can be explored through data mining by systematically evaluating activity annotations ^1^. For the assessment of compound and drug promiscuity, public databases such as ChEMBL ^4^, the major repository of compounds and activity data from medicinal chemistry, the PubChem BioAssay database ^5^, the major repository of screening data, and DrugBank ^6^, which collects approved and experimental drugs, have become indispensible resources.

Computational analyses reported thus far have suggested different degrees of promiscuity among bioactive compounds and drugs, dependent on the compound sources used and the methods applied. For example, drug-target network analysis has indicated that a drug might on average act on two targets ^7^. Other computational studies have suggested that drugs might on average interact with two to seven targets depending on the target classes the drugs are active against ^8^. In addition to varying compound sources and analysis concepts, taking activity measurement characteristics and data confidence criteria into account is also of critical importance for compound promiscuity analysis. For example, it has been shown that the increase in the number of compounds with activity against targets from different families in ChEMBL has mostly resulted from assay-dependent IC _50_ but not (assay-independent) K _i_ measurements (equilibrium constants) ^9^. In addition, by exclusively considering high-confidence activity data, it has been found that the majority of promiscuous bioactive compounds interact with two to five targets from the same target family, are predominantly active in sub-µM range, and display potency differences within one or two orders of magnitude against their targets ^10^. This represents a prevalent promiscuity profile among bioactive compounds. On the basis of high-confidence activity data, it has also been calculated that compounds from ChEMBL interact on average with one to two targets and compounds from PubChem confirmatory assays with two to three targets ^11^. By contrast, target annotation analysis has suggested that approved drugs interact on average with close to six targets, whereas experimental drugs (including candidates in clinical trials) interact with one to two targets ^11^. The reasons for this apparent discrepancy in target numbers between drugs at different development stages are currently unknown. As increasing amounts of activity data become available, it is likely that recently detected promiscuity rates might further increase. However, the magnitude of such increases as a consequence of data incompleteness ^12^ is difficult to predict, especially considering the low promiscuity rates that can currently be confirmed on the basis of high-confidence data ^1, 11^.

In this study, we further extend the computational analysis of promiscuity by evaluating the progression of drug promiscuity rates over time, which required a systematic assessment of activity records with release dates. Different data selection criteria were applied and the calculated promiscuity rates were compared to available drug target annotations. Small to moderate increases in drug promiscuity over time were detected when high-confidence activity data were considered. Lowering the stringency of data selection criteria led to unrealistic estimates of promiscuity rates and their progression.

## Materials and methods

### Data collection

From ChEMBL (release 18) ^4^, compounds with direct interactions (i.e., assay relationship type “D”) with human targets at the highest confidence level (i.e., assay confidence score 9) were collected. The two ChEMBL parameters ‘assay relationship type’ and ‘assay confidence score’ qualify and quantify the level of confidence that the activity against a given target is evaluated in a relevant assay system, respectively. Accordingly, type “D” and score 9 represent the highest level of confidence for activity data. In addition, two types of activity measurements were considered including assay-independent equilibrium constants (K _i_ values) and assay-dependent IC _50_ values. To ensure a high level of data integrity, only compounds with explicitly defined K _i_ or IC _50_ values were selected. Hence, approximate measurements such as “>”, “<”, and “~” were disregarded. Compounds with multiple K _i_ or IC _50_ measurements for the same target were retained if all these values fell within the same order of magnitude. Otherwise, the target activity was omitted from further consideration. Structures of all qualifying bioactive compounds were standardized using the Molecular Operating Environment (MOE) ^13^ and transformed into canonical SMILES strings ^14^. The so assembled compound set exclusively utilized high-confidence activity data (high-confidence data set).

Approved small molecule drugs with available structure and activity information were collected from the latest release of DrugBank (version 4.1) ^6^. To synchronize the activity analysis in ChEMBL and DrugBank, all reported ‘drug action’ targets, metabolizing enzymes, transporters, and carriers were assembled for approved drugs. In some instances, drug target activity might refer to a group of related proteins. For example, atomoxetine was annotated with N-methyl-D-aspartate (NMDA) receptor including seven subtypes. Accordingly, seven UniProt ^15^ accession IDs (UniProtIDs) were associated with NMDA receptor. Thus, the maximal number of target annotations was collected for approved drugs on the basis of UniProtIDs. Drug structures were also standardized using MOE and transformed into canonical SMILES strings.

### Monitoring drug activity records over time

**Figure 1.**
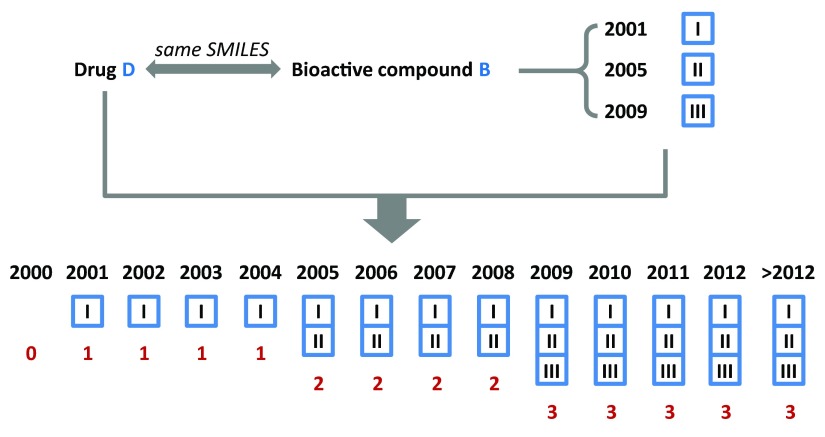
Organization of activity records. The organization of the activity records for a drug over different years is schematically illustrated. Drug D and a bioactive compound B share the same SMILES string (D is mapped to B). The activity records of compound B are extracted from ChEMBL. B is reported to be active against target I in 2001, II in 2005, and III in 2009. These activity records are then assigned to drug D and organized into 14 time intervals (12 of which represent individual years, except 2000 (see text) and >2012). For each interval, a cumulative activity profile is generated for D and recorded. The total number of activity annotations is given in red.

Most compound activity data in ChEMBL are extracted from medicinal chemistry literature and patent sources ^4^. Therefore, the release dates of activity data are frequently recorded in this database. However, DrugBank does not report dates for individual target annotations. To systematically monitor drug promiscuity over time, all approved drugs from DrugBank were mapped to ChEMBL by comparing canonical SMILES strings. If a drug (D) and a bioactive compound (B) shared the same SMILES string, a match was obtained. It should be noted that the name of a drug in DrugBank and ChEMBL might differ (i.e., matching by drug/compound name is not reliable). For each match, activity data release dates of compound B were recorded and assigned to drug D. Each activity record represented a target annotation (the terms target activity and target annotation are synonymously used). For instance, if compound B was reported to be active against target I in 2001, target II in 2005, and target III in 2009, the cumulative activity records for drug D consisted of target I in 2001, targets I and II in 2005, and targets I, II, and III in 2009. Thus, the promiscuity rate of D increased over time from 1 to 3. All activity records were organized into 14 time intervals, as illustrated in Figure 1. All activity records reported before 2000 were assigned to 2000, the starting point of our analysis, and all activity data released after 2012 were assigned to the last period “>2012”. For each time interval, the cumulative activity profile was recorded. Hence, changes in the promiscuity rate of a drug were successively determined over the years. Cumulative activity profiles were compared to target annotations available in DrugBank.

### Low-confidence data sets

In order to investigate the effect of activity data confidence levels on drug promiscuity, two data sets with lower confidence were assembled from ChEMBL (release 18). For the generation of low-confidence data sets, two criteria that influence the compound data integrity, i.e., the confidence level of activity and the type of activity measurements were disregarded in subsequent steps. In low-confidence set 1, the criterion of activity measurement type was not considered. Hence, in addition to K _i_ and IC _50_ values, all other potency annotations were equally considered (including “%max”, “Efficacy”, “EC _50_”, “K _d_”, and “Residual Activity”) for all compounds with ‘direct interactions’ with human targets and assay confidence score 9. In addition, the consistency and quality of potency measurements was not considered. In low-confidence set 2, the confidence level of activity (assay relationship type and assay confidence score) was not considered, in addition to the type of activity measurements. Therefore, the stringency of activity data and compound selection decreased from the high-confidence set over low-confidence set 1 to low-confidence set 2.

Progression of drug promiscuity over time was systematically evaluated on the basis of all three data sets.

## Results and discussion

### Bioactive compounds and approved drugs

On the basis of the selection criteria described above, a total of 143,424 bioactive compounds with high-confidence activity data were obtained from ChEMBL. These compounds were active against 1376 different targets and yielded 219,602 compound-target interactions, as reported in Table 1. Furthermore, from DrugBank 4.1, 1429 approved drugs were obtained that were annotated with 1657 target proteins corresponding to 10,679 drug-target interactions ( Table 1). Thus, there were nearly 100 times more bioactive compounds than approved drugs. However, with 1657 targets, drugs covered a larger target space than bioactive compounds (1376 targets). On average, a bioactive compound was active against 1.5 targets, whereas an approved drug was annotated with 7.5 targets. Compared to a recent analysis of promiscuity rates ^11^, which also included a previous release of DrugBank, the average promiscuity rate of approved drugs further increased from 5.9 to 7.5, while the degree of promiscuity among bioactive compounds remained essentially constant.

**Table 1.** Data sets.

Number of	DrugBank 4.1	ChEMBL release 18
**Drugs/compounds**	1429	143,424
**Targets**	1657	1376
**Interactions**	10,679	219,602

For DrugBank 4.1 (drugs) and ChEMBL release 18 (compounds), the number of drugs/compounds, targets the drugs/compounds were active against, and the total number of interactions is reported.

To monitor drug promiscuity over time, all approved drugs were mapped to bioactive compounds in ChEMBL for which release dates of activity records were reported (as detailed in the Methods section). For 518 of the 1429 approved drugs taken from DrugBank, high-confidence activity data released over different years were found in ChEMBL. These 518 drugs provided the basis for our time-dependent promiscuity analysis.

### Data inconsistency

**Figure 2.**
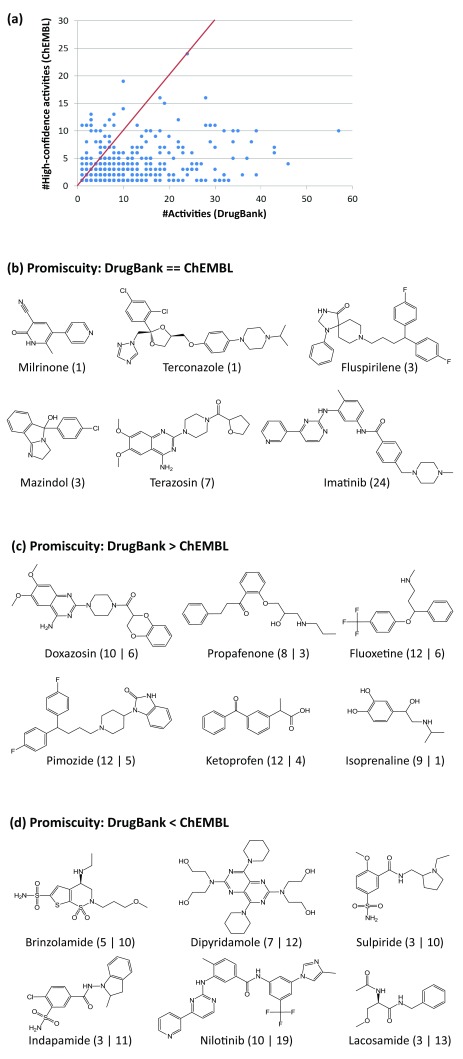
Drug promiscuity in DrugBank vs ChEMBL. ( **a**) For 518 qualifying drugs, the number of targets reported in DrugBank and the number of high-confidence activity annotations in ChEMBL are compared in a scatter plot. Each dot represents a drug. The diagonal (indicating perfect correlation) is drawn in red. In ( **b**), ( **c**), and ( **d**), exemplary drugs are shown that had the same number of targets in DrugBank and ChEMBL (i.e., the same promiscuity rate), a higher promiscuity rate in DrugBank, and a higher rate in ChEMBL, respectively. For each drug in ( **b**), the number of targets is given. For each drug in ( **c**) and ( **d**), the numbers of targets reported in DrugBank and ChEMBL are compared. For example, “10 | 6” indicates that the drug was annotated with 10 targets in DrugBank and with six in ChEMBL.

For the 518 qualifying drugs, we first compared their target annotations in DrugBank and the total number of targets derived from high-confidence activity records in ChEMBL. As reported in Figure 2a, most of the drugs had different numbers of targets in the two databases. Only 32 drugs (~6%) were found to have the same number of target annotations in DrugBank and ChEMBL. The total number of target annotations of a drug represented its promiscuity rate. A total of 439 drugs had higher promiscuity rates in DrugBank than in ChEMBL. Opposite observations were only made for 47 drugs. On average, the 518 drugs were annotated with ~10.1 targets in DrugBank and ~3.2 targets derived from high-confidence ChEMBL activity records. Hence, promiscuity rates in DrugBank were much higher than in ChEMBL. Exemplary drugs having the same or different degrees of promiscuity in DrugBank and ChEMBL are shown in Figure 2b– Figure 2d.

**Figure 3.**
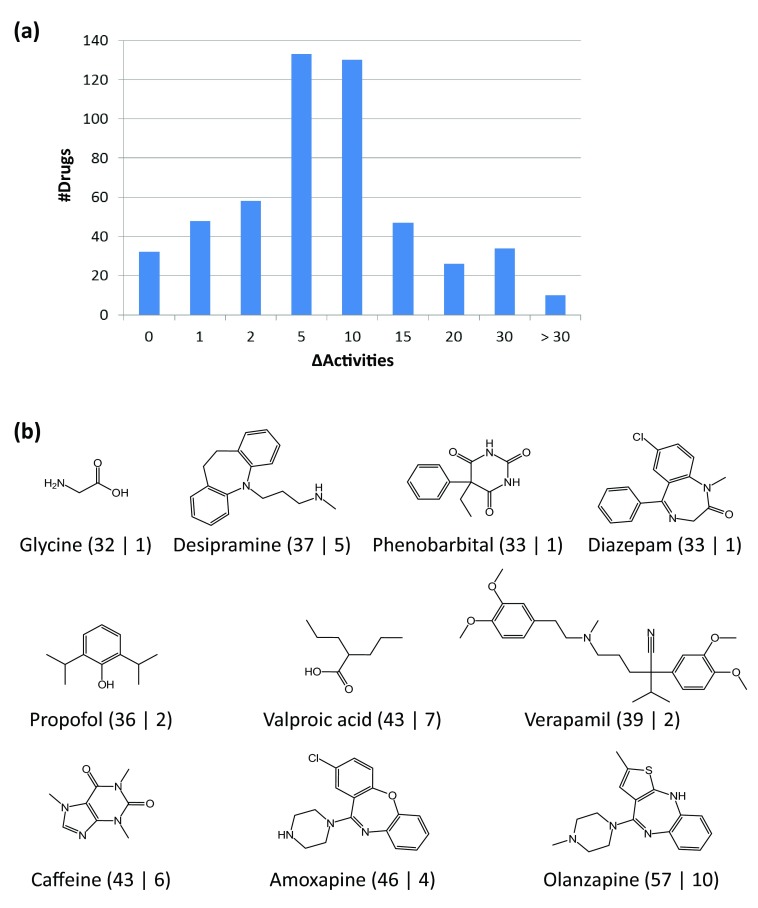
Promiscuity variation in DrugBank vs ChEMBL. ( **a**) Reported is the distribution of promiscuity differences (∆Activities) between DrugBank and ChEMBL for 518 drugs. ( **b**) Shown are 10 drugs with the largest difference in promiscuity (∆Activities > 30). Target annotations are represented according to Figure 2c and Figure 2d.

Differences in promiscuity rates were quantified, as reported in Figure 3a. Among the 486 drugs (~94%) with varying degrees of promiscuity in DrugBank and ChEMBL, 48 and 58 drugs differed by one and two targets, respectively. By contrast, the promiscuity rates of nearly half of the drugs (247; ~48%) varied by more than five targets. Moreover, for the 10 drugs shown in Figure 3b, the promiscuity rates differed by more than 30 targets, which reflected a particularly high degree of data inconsistency. All of these drugs were annotated with many more targets in DrugBank than targets derived from high-confidence activity records in ChEMBL. The extreme case was olanzapine the promiscuity rate of which differed by 47 targets between the two databases.

**Figure 4.**
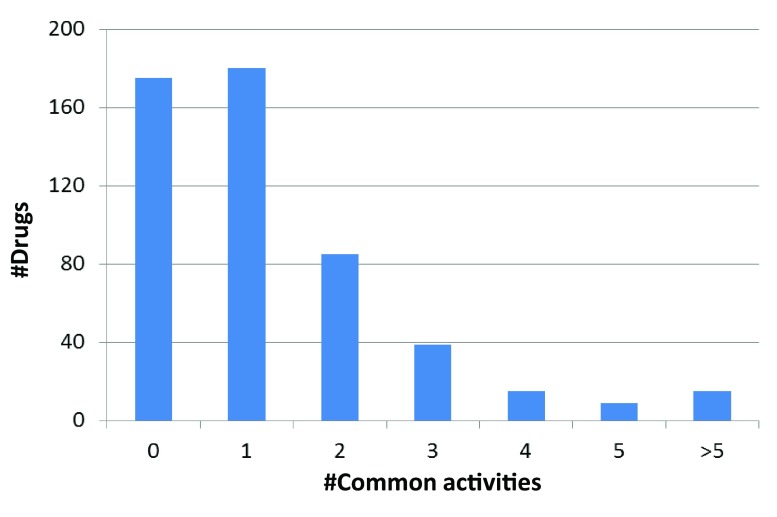
Comparison of activity profiles in DrugBank and ChEMBL. The activity profiles of 518 drugs in DrugBank and ChEMBL are compared. Reported is the number of drugs sharing increasing numbers of activities (#Common activities) in the two databases.

In addition to comparing the number of target annotations, the activity profiles of drugs were further examined to determine the consistency of the annotations. As reported in Figure 4, 175 drugs (~34%) had non-overlapping sets of targets in these two databases, which was another surprising finding. The remaining 343 drugs had overlapping yet distinct target sets. However, the majority of these drugs shared only one or two targets, reflecting substantial discrepancies between target annotations.

For the study of changes in drug promiscuity over time, accessing original activity records and their release dates was an essential requirement, as rationalized above. Such information is not available in DrugBank.

### Drugs on a time course

**Figure 5.**
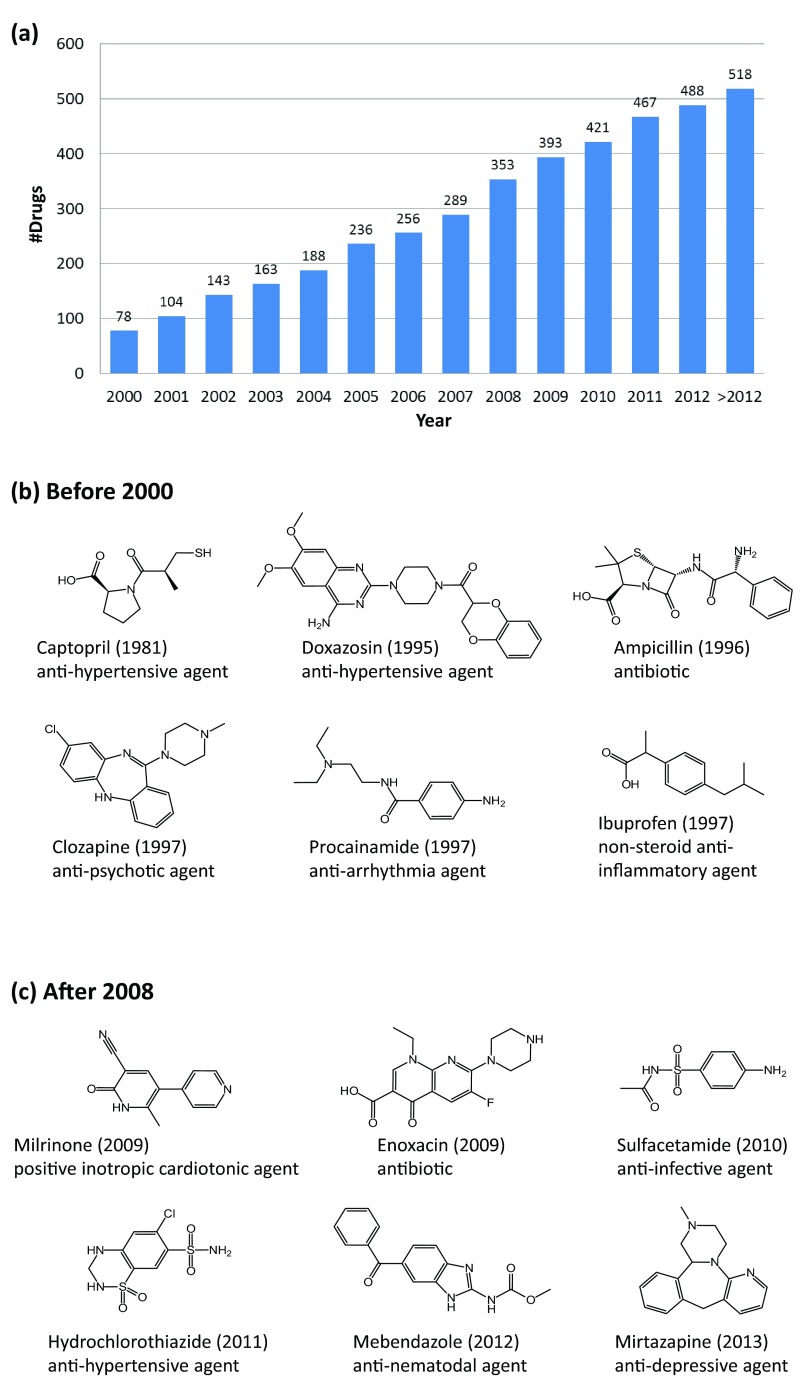
Monitoring high-confidence drug activity data over time. ( **a**) Reported is the cumulative number of drugs for which high-confidence activity data became available in different years. In ( **b**) and ( **c**), six exemplary approved drugs are shown for which high-confidence activity data were first recorded before 2000 or after 2008, respectively. For each drug, its name, year of first data report, and therapeutic indication are provided.

Next, we organized the 518 drugs on the basis of activity record release dates. Drugs were assigned to the individual time intervals in which high-confidence activity data were first published. For example, if the first activity record of a given drug was detected in 2005, the drug was assigned to the 2005 interval and traced during all subsequent years. The cumulative number of drugs in different time intervals is reported in Figure 5a. By 2000, high-confidence activity data were publicly available for 78 drugs. From 2000 to 2001, activity data became available for 26 additional drugs. The number of drugs for which qualifying activity records were available in subsequent years ranged from 20 to 64, with an average of ~34 drugs per interval. The largest increase was detected for 2007/2008. The time period for which the activity records were assembled spanned a maximum of 24 years (for captopril, from 1981 to 2005), with an average of 3.3 years per drug. Exemplary drugs for which activity records were first reported before 2000 and after 2008 are shown in Figure 5b and Figure 5c, respectively.

### Changes in drug promiscuity over time

**Figure 6.**
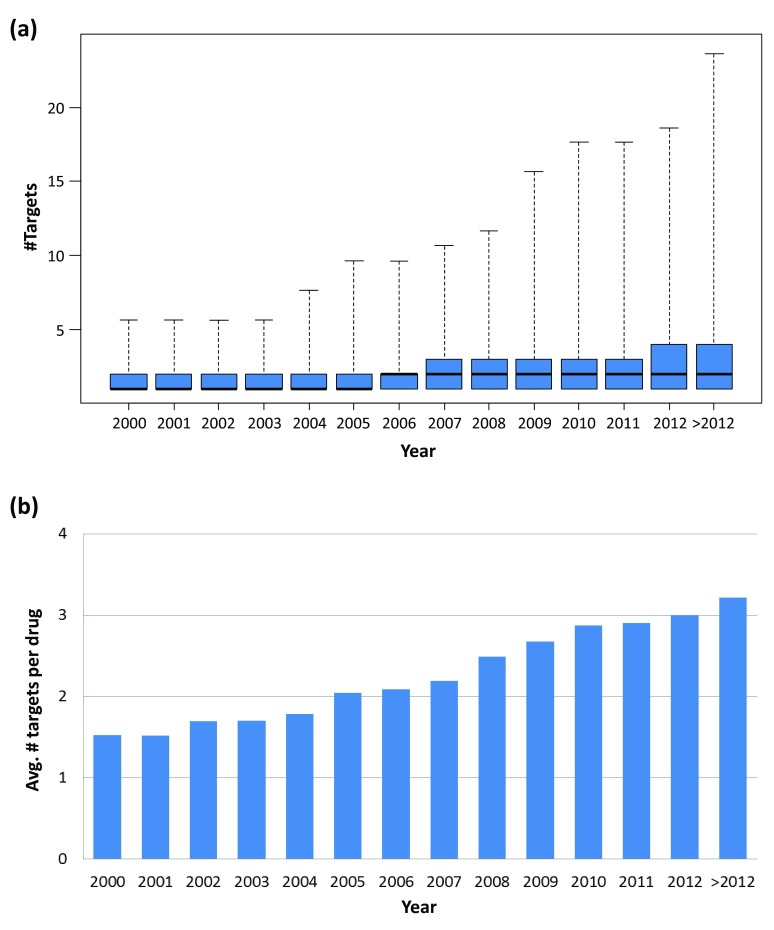
Monitoring drug promiscuity over time. ( **a**) Box plots capture the distribution of the number of targets per drug in different years. Each box plot reports the smallest value (bottom line), lower quartile (lower boundary of the box), median (thick horizontal line), upper quartile (upper boundary of the box), and the largest value (top line). ( **b**) Reported are average numbers of targets per drug in different years.

For individual time intervals, the distribution of drug promiscuity rates was determined, as reported in Figure 6a. The box plots reveal an increase in drug promiscuity rates over time, with a maximal rate of six targets per drug in 2000 and 24 targets per drug in interval >2012. However, median promiscuity rates only slightly increased from one (until 2005) to two (beginning in 2006) targets per drug. The distribution of average promiscuity rates is shown in Figure 6b, which slightly but steadily increased over time from 1.5 to 3.2 targets per drug. The larger relative increase of average than median promiscuity rates indicated that the average values were influenced by small numbers of drugs with large numbers of targets, i.e., a small subset of highly promiscuous drugs, consistent with earlier observations ^11^. On the basis of median values, detectable increases in drug promiscuity over time were limited.

**Figure 7.**
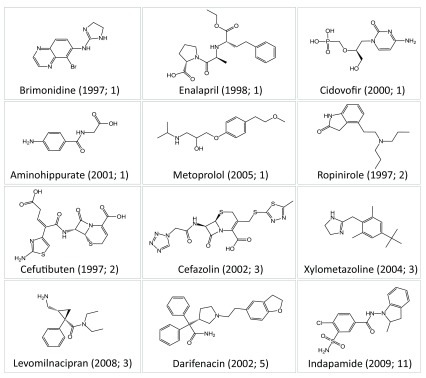
Drugs with constant promiscuity over time. Shown are 12 exemplary drugs having a constant promiscuity rate on the basis of high-confidence activity data. For each drug, the year of its first activity report and the number of targets it was active against are given. For example, brimonidine was first reported to be active against a single target in 1997.

**Figure 8.**
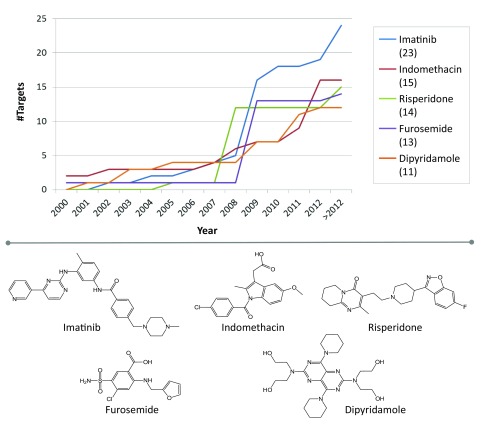
Top five drugs with largest changes in promiscuity. For the five drugs with largest changes in promiscuity over time, cumulative numbers of targets are reported for different years (top). For each drug, the overall difference in target annotations is given in parentheses. The structures of these drugs are shown at the bottom.

Changes in promiscuity over time were also monitored for individual drugs. For each drug, the increase in the cumulative promiscuity rates from its first to its most recent activity records was determined (for the hypothetical example in Figure 1, the increase in promiscuity rates is 2). For the 518 drugs, increases are reported in Table 2. Surprisingly, for 282 drugs (~54%), no increase in promiscuity was detected on the basis of high-confidence activity records. This indicated that the majority of these drugs did not receive additional high-confidence activity annotations since their first records were released. Of 282 drugs, 203 drugs were only annotated with a single target. Exemplary drugs with constant promiscuity rates are shown in Figure 7. For the remaining 236 drugs, increasing numbers of targets were detected. However, in most cases, the increase in target numbers was limited, i.e., the promiscuity rates of 197 drugs increased by one to five targets ( Table 2). There were only 14 drugs with an increase in promiscuity rates by 10 or more targets. Five drugs with largest increase in promiscuity rates are shown in Figure 8. For example, the promiscuity rate of imatinib increased from one in 2002 to 24 (>2012), with 11 new targets reported between 2008 and 2009. The drugs in Figure 8 belonged to the subset of highly promiscuous drugs that statistically influenced the calculation of average promiscuity rates, as discussed above.

**Table 2.** Increasing promiscuity.

Increase in promiscuity rates	#Drugs (%)
**0**	282 (54.4%)
**1**	84 (16.2%)
**2**	44 (8.5%)
**3**	39 (7.5%)
**4**	15 (2.9%)
**5**	15 (2.9%)
**6**	5 (1.0%)
**7**	8 (1.5%)
**8**	6 (1.2%)
**9**	6 (1.2%)
**10**	8 (1.5%)
**> 10**	6 (1.2%)

The number (percentage) of drugs with increasing promiscuity rates (i.e., number of targets) is reported.

Drug promiscuity across different target families was also assessed. For the 236 drugs with increases in promiscuity rates over time, their targets were assigned to families and the number of target families was determined and followed over time. Table 3 reports the number of drugs with increasing target family annotations. For the majority of drugs, the number of target families increased by one or two. For top five drugs with largest changes in promiscuity ( Figure 8), their target family profiles are provided in Table 4. The first activity records of all these five drugs belonged to only one target family including protein kinase family, GPCR subfamily, and transporter subfamilies. Compared to their most recent activity records, the number of target families increased by three to nine, spanning a wide range of related or unrelated target families. It indicated that these drugs might have been tested against a large panel of targets over time and that a number of activities have been confirmed at a high level of confidence. For 47 drugs, the number of target families remained constant.

**Table 3.** Promiscuity increase across different target families.

Increase in promiscuity across target families	#Drugs (%)
**0**	47 (19.9%)
**1**	105 (44.5%)
**2**	47 (19.9%)
**3**	21 (8.9%)
**4**	9 (3.8%)
**5**	4 (1.7%)
**> 5**	3 (1.3%)

The number (percentage) of drugs with increasing target family promiscuity (i.e., increasing number of protein families the drug targets belong to) is reported.

**Table 4.** Target family profiles for top five drugs with largest changes in promiscuity.

Drug name	#Targets	#Families	Family list
Imatinib	24	7	ATP binding cassette transporters; Carbonic anhydrases; Multi antimicrobial extrusion (MATE) transporters; NAD(P)H dehydrogenases (quinone); Organic cation transporters; Ser_Thr protein kinases; **Tyr protein kinases**
Indomethacin	16	10	ATP binding cassette transporters; Aldo/keto reductases; Glyoxalases I; Intercrines; **Lipoxygenases**; MAPEGs; Organic cation transporters; Organo anion transporters; Potassium ion channels; Short-chain dehydrogenases/reductases (SDR)
Risperidone	15	4	**Monoamine GPCRs**; Multi antimicrobial extrusion (MATE) transporters; Organic cation transporters; Sodium:neurotransmitter symporters (SNF)
Furosemide	14	4	**Bile acid:sodium symporters (BASS)**; Carbonic anhydrases; Carboxylic acid GPCRs; Short-chain dehydrogenases/reductases (SDR)
Dipyridamole	12	5	**ATP binding cassette transporters**; Multi antimicrobial extrusion (MATE) transporters; Organic cation transporters; Class C PPases; Phosphodiesterases

For the top five drugs with largest changes in promiscuity over time, the total number of targets and families is reported. The family with the first target annotation of a drug is shown in bold. Target family abbreviation: MAPEG, membrane-associated proteins in eicosanoid and glutathione metabolism.

### Drug promiscuity on the basis of low-confidence data sets

**Figure 9.**
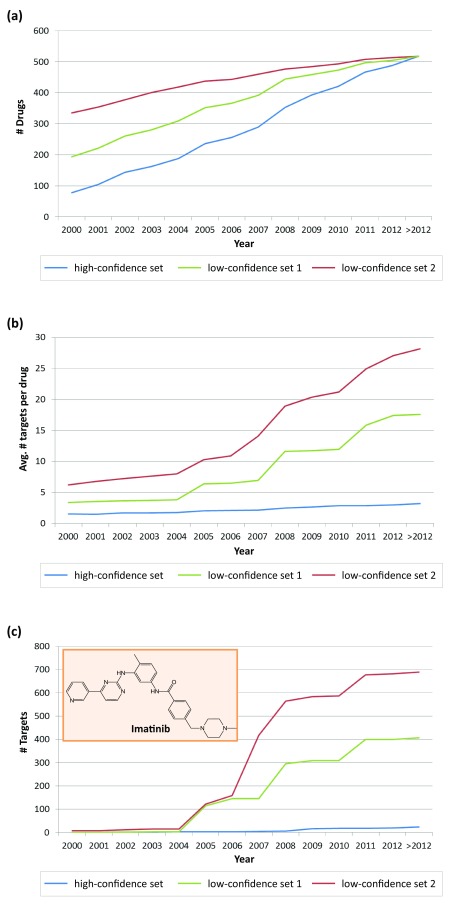
Drugs in sets with varying activity confidence levels. ( **a**) Reported is the cumulative number of drugs in three data sets of varying confidence levels over time. ( **b**) Shown is the distribution of average promiscuity rates for drugs in these three data sets. ( **c**) For imatinib, the cumulative number of targets is reported for different years.

Two compound sets with lower activity data confidence were also assembled from ChEMBL, as described above. The composition of these sets is summarized in Table 5. Low-confidence set 1 in which the types of activity measurements were not specified contained a total of 605,206 compounds active against 2144 targets, yielding more than 2,600,000 interactions. Low-confidence set 2 in which, in addition, the confidence level of activity was undefined consisted of a larger number of 936,924 compounds active against 3934 targets, yielding more than 6,000,000 interactions. All 518 drugs were mapped to these two low-confidence data sets. The cumulative distribution of these drugs over time is reported in Figure 9a. The number of drugs with low-confidence activity annotations in 2000 increased from 78 (high-confidence set) to 194 (low-confidence set 1) and 335 (low-confidence set 2). On average, ~26 and ~15 drugs became available during each year for low-confidence set 1 and 2, respectively.

**Table 5.** Low-confidence data sets.

Number of	Set 1	Set 2
**Compounds**	605,206	936,924
**Targets**	2144	3934
**Interactions**	2,639,767	6,295,086

For both low-confidence sets (see text for details), the number of compounds, targets, and interactions is reported.

Figure 9b compares the distribution of average drug promiscuity rates for the three data sets over time. In contrast to the high-confidence data set in which drug promiscuity only slightly increased over the years, the average promiscuity rates of drugs in both low-confidence sets were higher and significantly increased. In low-confidence set 2, the average promiscuity rate was 6.3 targets per drug in 2000 and further increased to 28.2 targets (>2012). Thus, by reducing the stringency of selection criteria for activity records, high average promiscuity rates were obtained. The large increases in average promiscuity rates seen in Figure 9b ultimately resulted in 18 (low-confidence set 1) or nearly 30 (set 2) targets per drug are most likely artificial in nature. The comparison reveals how the choice of different activity data selection criteria, or the lack of well-defined criteria, might bias promiscuity analysis.

Imatinib represented a striking example for the presence of unreliable target annotations under non-stringent data selection criteria ( Figure 9c). In both low-confidence sets 1 and especially 2 dramatic increases were observed between 2005 and 2008, ultimately leading to 406 and 689 targets for imatinib, respectively (hence exceeding the total number of targets in the human kinome). By contrast, on the basis of high-confidence activity data, the final (>2012) promiscuity rate of imatinib was 24.

**Figure 10.**
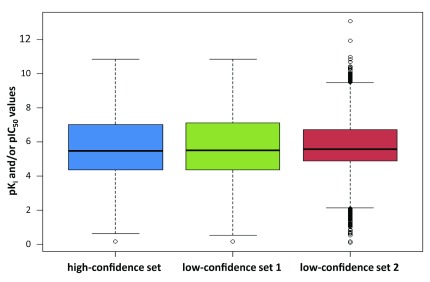
Potency distribution. The distribution of pK _i_ and/or pIC _50_ values in three data sets with varying confidence levels is reported in box plots. Each box plot provides the lowest potency value within the 1.5 interquartile range of the lower quartile (bottom line), lower quartile (lower boundary of the box), median value (thick line), upper quartile (upper boundary of the box), and the highest value within the 1.5 interquartile range of the upper quartile (top line). Potency values falling outside these ranges are indicated by empty circles.

In addition, the distributions of potency values were compared across different data sets, as reported in Figure 10. It should be noted that only K _i_ and IC _50_ values were considered here, although all other types of potency annotations were included in low-confidence sets 1 and 2. In general, the distribution of the high-confidence set was comparable to the low-confidence set 1. The majority of negative logarithmic potency values ranged from ~4.5 (i.e., ~32 µM) to 7.0 (i.e., 100 nM). By contrast, the majority of potency values in low-confidence set 2 were confined to a narrow range.

### PAINS substructures

**Figure 11.**
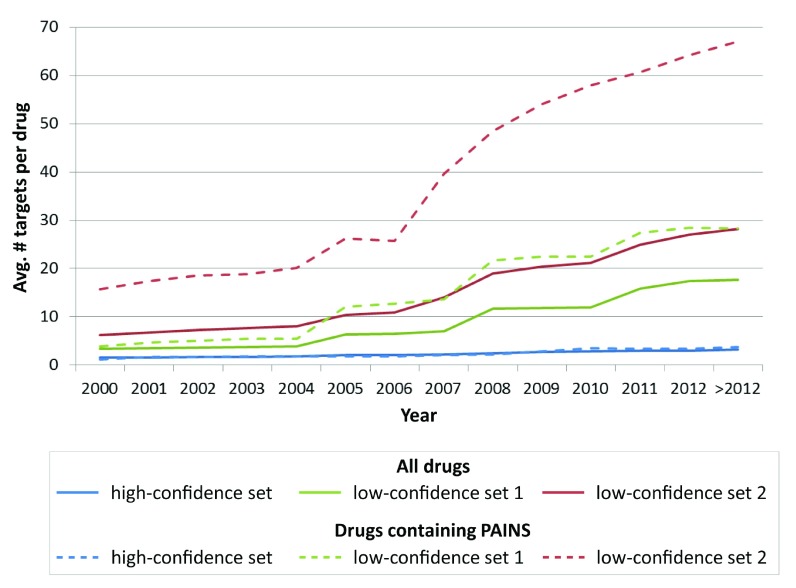
Drugs containing PAINS substructures. Shown is the distribution of average promiscuity rates over time for all drugs (solid lines) and for drugs that contain PAINS substructures (dashed lines) in three data sets with varying activity confidence levels, respectively.

Compounds that are reactive or cause other non-specific effects in a variety of assays are typically false positives and have been termed pan assay interference compounds (PAINS) ^16^. Baell and Holloway described a set of 26 substructures that are indicative of PAINS liability) ^16^. This set of substructures was utilized as a filter to identify drugs that contain PAINS substructures in our three data sets with varying activity confidence levels. A total of 23 drugs (i.e., ~4.4%) were found to contain PAINS substructures. Figure 11 reports the average promiscuity rates of PAINS-positive drugs compared to all available drugs over time. It can be seen that drugs with potential PAINS liability in two low-confidence sets displayed much higher degrees of promiscuity than the global rates. For example, the latest average promiscuity rate (i.e., >2012) of drugs containing PAINS substructures in low-confidence set 2 increased from ~28.2 to ~67.0. By contrast, PAINS-positive drugs in the high-confidence set displayed a comparable degree of promiscuity. These findings suggest that PAINS-related effects might also be controlled by applying rigorous data confidence criteria.

## Conclusions

The analysis reported herein was designed to monitor drug promiscuity over time through computational data mining. It was facilitated by systematically collecting available activity records with release dates for approved drugs from the ChEMBL database. For more than 500 drugs, it was possible to assess promiscuity rates over a time course. Current promiscuity rates derived from high-confidence ChEMBL activity records are typically much lower than those calculated from target annotations available in DrugBank, which should merit further consideration. Data selection criteria for the assignment of drug targets might at least in part be responsible for the observed differences. On the basis of high-confidence activity data, an increase in the average drug promiscuity rates from only 1.5 to 3.2 targets per drug was observed. The magnitude of average promiscuity rates was influenced by a small subset of highly promiscuous drugs. Thus, increases in average drug promiscuity over time were generally small. However, they frequently involved targets from at least two families. By contrast, for low-confidence data sets, calculated promiscuity rates were much higher and dramatic increases in apparent drug promiscuity were observed over the years. From our point of view, such trends are unreliable. These observations further emphasize the need for well-defined and stringent data selection criteria for promiscuity analysis. Taken together, the findings reported herein reveal a small to moderate increase in detectable drug promiscuity over time while the volumes of compound activity data rapidly grow.

## Data availability

The high-confidence and the two low-confidence drug data sets are made available in ZENODO. For each drug in each set, the ChEMBL activity records are provided for individual time intervals.

ZENODO: Drug activity data, doi: 10.5281/zenodo.11576 ^17^
