# Supplementary material for: Monitoring drug promiscuity over time
Source: F1000Res. 2014 Nov 4;3:218. Originally published 2014 Sep 11. [Version 2] doi: 10.12688/f1000research.5250.2 (PMC4207249; doi:10.12688/f1000research.5250.2)
Supplement: Peer review history

# Peer review: Referee response for version 1

**DOI:** 10.5256/f1000research.5597.r6405

**Published:** 2014-10-24

**Authors:** Michael Walters, Kathy Nelson

**Affiliations:** 1 Department of Medicinal Chemistry, University of Minnesota, Minneapolis, USA; 2 University of Minnesota, Minneapolis, USA

The authors have presented an interesting and careful study on drug promiscuity as analyzed through available activity databases. The authors also illustrate the different conclusions that can be drawn based on the quality of the data analyzed. The discrepancies they point out highlight the importance of high-quality data when evaluating the promiscuity of drugs.

The manuscript is clear and well-written, the methods are scientifically sound, and the analysis is thorough and clearly presented.

**Comments:**

*“Compounds with multiple K _i_ or IC _50_ measurements for the same target were retained if all these values fell within the same order."* Should this read “…the same order of magnitude.”?

*“Surprisingly, for 282 drugs (~54%), no increase in promiscuity was detected on the basis of high-confidence activity records.*” It would increase the impact of this article if the authors explained why this finding is surprising.  For example, do these compounds represent drugs that were highly optimized for a single target?

*“There were only 14 drugs with an increase in promiscuity rates by 10 or more targets.“* As above, the authors might want to add their thoughts as to why these drugs have such high promiscuity rates.  For example, are they kinase inhibitors which had known broad kinase panel activity and the complete scope of their kinase activity is only now coming to light?

**Suggestions for minor modifications:**

The dataset has been made publicly available through a DOI link. The link was broken when I attempted to follow it and look at the data. This link should be fixed.

The authors have not provided any summary of the criteria used to determine what constitutes activity of a compound at a target (e.g. 10 μM cut-off? 100 μM?). If a strict cut-off cannot be given for each dataset, it is suggested that authors reported the range of activities reported in each dataset (one for the high-confidence data set, and one for each of the two low-confidence data sets).

The authors mention that all the compounds were converted into SMILES strings during data analysis. It is suggested that the authors screen the SMILES list against a REOS/PAINS/etc. filters. It would be interesting to see if any of the high promiscuity compounds could be flagged as having substructures that could be responsible for non-specific activity. Or would have impeded the development of these drugs had they been prepared after cheminformatic filtering came into vogue.

These suggested modifications would help increase the impact of this manuscript and could be of great interest to the drug discovery community.

We have read this submission. We believe that we have an appropriate level of expertise to confirm that it is of an acceptable scientific standard.

# Peer review: Referee response for version 1

**DOI:** 10.5256/f1000research.5597.r6398

**Published:** 2014-10-16

**Authors:** Stefan Laufer

**Affiliations:** 1 Pharmaceutical Chemistry, Eberhard Karls University Tubingen, Tubingen, Germany

Key points:

1. 500+ drugs successfully mapped to ChEMBL to follow a time course of promiscuity.
2. When high-confidence activity data were considered, only small average increase in drug promiscuity over time were observed (from ca 1.5-3).
3. When the stringency of data selection criteria was gradually relaxed and low-confidence data were considered, unrealistic increases in promiscuity over time were detected.
4. As an illustration, please, see the imatinib example in Figure 9 (in comparison to Figure 8a) that the reader might find interesting.

In addition, please, see the conclusions, especially the second part beginning with " On the basis of high- confidence activity data ..."

I have read this submission. I believe that I have an appropriate level of expertise to confirm that it is of an acceptable scientific standard.

# Peer review: Referee response for version 1

**DOI:** 10.5256/f1000research.5597.r6235

**Published:** 2014-10-15

**Authors:** Steffen Renner

**Affiliations:** 1 Novartis Institute for Biomedical Research, Basel, Switzerland

The authors provide and interesting study on drug promiscuity. Increasing our understanding of this important topic is of high value for the advancement of drug discovery.  The current study investigates drug promiscuity over time, i.e. the number of targets reported for drugs over time. The authors conclude that this number is surprisingly low, if only high quality interactions are considered.

The manuscript is well written, scientifically sound, and the analyzed dataset is made publicly available.

Some points for minor modifications:

 

- When I read the title my first impression was that the manuscript compares the promiscuity of drugs with respect to their release data, e.g. "do more recent drugs show more or less promiscuity than older drugs". I suggest to change the title to better reflect that the release date of the target information is analyzed rather than the release date of the drugs.
- Page 4, second column: The only definition of the promiscuity rate I found in the manuscript is the following: "The total number of target annotations of a drug represented its promiscuity rate". The Wikipedia definition of a rate is "Rate (mathematics), a specific kind of ratio, in which two measurements are related to each other", therefore it appears to me that another name might better reflect the meaning of the promiscuity rate, e.g. just "promiscuity". On the other hand it is not the promiscuity which is changing: it is the number of identified interactions. Therefore another name might be even better.
- I did not find an activity cut-off (e.g. 10μM?) used for the activities in ChEMBL. Please add this information.

I have read this submission. I believe that I have an appropriate level of expertise to confirm that it is of an acceptable scientific standard.

# Response

**Published:** 2014-10-20

**Authors:** Jürgen Bajorath

**Affiliations:** Department of Life Science Informatics, B-IT and LIMES Institutes, Rheinische Friedrich-Wilhelms-Universität Bonn, Germany

We thank the reviewer for his comments and the points raised.

1. "Title": We would prefer retaining the current concise title. In our view, it reflects the time course of the promiscuity analysis.
2. "Rate": From a mathematical/statistical point of view, the reviewer is correct ("rate" reflects a ratio). However, outside statistics, the term "rate" is often used synonymously with "degree", which is the intended meaning here. In previous publications, we have used both terms in the context of promiscuity analysis. We agree that the use of the term "degree" would avoid a potential inconsistency and should be preferred.
3. "Activity cut-off": A cut-off value has not been applied in this analysis because the promiscuity time course was compared on the basis of low- vs. high-confidence activity data. The preferred use of high-confidence data would make the application of activity cut-off values a matter of debate. In a previous study (reference 11), it was shown that representative promiscuous compounds identified on the basis of high-confidence activity data are rarely weakly potent against their targets (which we consider an interesting observation).

Many thanks again to both referees for taking the time to review this manuscript
